# Association between different risk factors and vascular accelerated ageing (EVA study): study protocol for a cross-sectional, descriptive observational study

**DOI:** 10.1136/bmjopen-2016-011031

**Published:** 2016-06-07

**Authors:** Manuel A Gomez-Marcos, Carlos Martinez-Salgado, Rogelio Gonzalez-Sarmiento, Jesus Ma Hernandez-Rivas, Pedro L Sanchez-Fernandez, Jose I Recio-Rodriguez, Emiliano Rodriguez-Sanchez, Luis García-Ortiz

**Affiliations:** 1 Primary Care Research Unit, Instituto of Investigación Biomédica of Salamanca (IBSAL), The Alamedilla Health Center, Castilla and León Health Service–SACYL, Salamanca, Castile and León, Spain; 2 Department of Medicine, REDIAPP, University of Salamanca, Salamanca, Castile and León, Spain; 3 Unit Renal Physiology and Pathophysiology Cardiovascular Unit, Department of Physiology and Pharmacology, IBSAL, Queen Sofia Institute of Nephrology Research, University of Salamanca, Salamanca, Castile and León, Spain; 4 IBSAL and Instituto of Biología Molecular and Celular of Cáncer (IBMCC), University of Salamanca–SACYL, Salamanca, Castile and León, Spain; 5 Department of Medicine, University of Salamanca, Castile and León, Spain; 6 IBSAL, IBMCC, Cancer Research Center, University of Salamanca, CSIC, University Hospital of Salamanca, Salamanca, Castile and León, Spain; 7 Department of Hematology, University of Salamanca, Salamanca, Castile and León, Spain; 8 Department of Medicine, University of Salamanca, Salamanca, Castile and León, Spain; 9 IBSAL, University Hospital of Salamanca, Salamanca, Castile and León, Spain; 10 Cardiology Department, University of Salamanca, Salamanca, Castile and León, Spain; 11 Primary Care Research Unit, IBSAL, The Alamedilla Health Center, Castilla and León Health Service–SACYL, REDIAPP, Salamanca, Castile and León, Spain; 12 Primary Care Research Unit, BSAL, The Alamedilla Health Center, Castilla and León Health Service–SACYL, Salamanca, Castile and León, Spain; 13 Biomedical and Diagnostic Sciences Department, REDIAPP, University of Salamanca, Salamanca, Castile and León, Spain

**Keywords:** Arterial stiffness, Aging, Physical activity, Psychological factors, Diet, Inflammatory markers

## Abstract

**Introduction:**

The process of population ageing that is occurring in developed societies represents a major challenge for the health system. The aim of this study is to analyse factors that have an influence on early vascular ageing (EVA), estimated by carotid-femoral pulse wave velocity (cf-PWV) and Cardio Ankle Vascular Index (CAVI), and to determine differences by gender in a Spanish population.

**Methods and analysis:**

An observational, descriptive, cross-sectional study.

**Study population:**

From the population assigned to the participating healthcare centres, a cluster random sampling stratified by age and gender will be performed to obtain 500 participants aged between 35 and 75. Those who meet the inclusion criteria and give written informed consent will be included in the study.

**Measurements:**

Main dependent variables: cf-PWV determined using the SphygmoCor System and CAVI estimated using VASERA. Secondary dependent variables: telomere length, carotid intima-media thickness, central and peripheral augmentation index, ankle-brachial pulse wave velocity, ankle-brachial index, retinal arteriovenous index, and renal and cardiac organ damage. Independent variables: lifestyles (physical activity, adherence to the Mediterranean diet, alcohol and tobacco consumption); psychological factors (depression, anxiety and chronic stress); inflammatory factors and oxidative stress.

**Ethics and dissemination:**

The study has been approved by the clinical research ethics committee of the healthcare area of Salamanca. All study participants will sign an informed consent form agreeing to participate in the study in compliance with the Declaration of Helsinki and the WHO standards for observational studies. The results of this study will allow the understanding of the relationship of the different influencing factors and their relative weight in the development of EVA. At least 5 publications in first-quartile scientific journals are planned.

**Trial registration number:**

NCT02623894; Pre-results.

**Strengths and limitations of this study**

- This study selected, by random sampling of the population, participants aged between 35 and 75 years.
- Assesses arterial ageing by the pulse wave velocity and Cardio Ankle Vascular Index (CAVI) arterial stiffness tests.
- Analyses the influence of diet, exercise, smoking and psychological factors on early vascular ageing (EVA).
- Assesses the influence of molecules of inflammation and oxidative stress in EVA.
- Analyses the telomere length and its association with vascular stiffness.

## Introduction

The process of population ageing that is occurring in developed societies represents a major challenge for the health system. Cardiovascular diseases (CVD) are the most common cause of morbidity and mortality in the world, representing half of the deaths in persons aged over 65 years and affecting >70% of patients with diabetes mellitus. The non-modifiable cause of vascular ageing in healthy adults is age, because it causes increased arterial stiffness and changes in chromosome replication with telomere shortening.^1^ ^2^

The comparison of biological age with chronological age allows us to establish if the pattern of vascular ageing is at an expected or accelerated rate, that is, if early vascular ageing (EVA) is present or not.^1^ ^2^

*Arterial stiffness and ageing*: Arterial stiffness increases with age even in healthy adults.^3^ It involves changes in vascular structure with thickening of the arterial wall, changes in vascular function due to increased stiffness, and decreased wall distensibility of large arteries, associated with endothelial dysfunction and pulse wave changes.^3^ ^4^ These changes are primarily mediated by nitric oxide.^1^ Arterial ageing is a multifactorial process associated with changes in structure and function of the large arteries.^1^ These changes are related to age, and are accelerated in the presence of associated CVD,^5–9^ cardiovascular risk factors or cardio-unhealthy lifestyles.^10^ Some of these factors are controllable, and a greater understanding of the links existing between vascular ageing, lifestyle and CVD will help find strategies geared to preventing or delaying this ageing.^11^ ^12^

*Telomere length and vascular ageing*: Somatic ageing starts when telomerase activity decreases and prevents accurate copies of chromosome replication being made in the distal end, resulting in a decreased telomere length.^13^ Although telomerase activity is genetically determined, it is also sensitive to the action of external factors.^1^ In addition to ageing, inflammation and oxidative stress are endogenous factors causing telomere shortening.^14^ Telomere length is also correlated with lifestyle.^14^ A reduced activity genotype causes a greater degree of vulnerability to triggering factors such as sedentary lifestyle, hypertension, obesity, hyperlipidaemia, diabetes, stress and smoking.^13^ The intimate relationship between the action of telomerase and morbidity and mortality has been documented in different studies.^13^ Rode *et al*^15^ analysed 64 637 individuals from the general population and concluded that short telomeres in peripheral blood leucocytes were associated with high mortality for individuals in the short test versus the longest decile. In 1525 postmenopausal women with a follow-up of 13 years, Carty *et al*^16^ found that white women with a shorter leucocyte telomere length (LTL) had higher risks of mortality and coronary heart disease. In contrast, shorter LTL was weakly associated with decreased mortality hazard in African-American women. Simpson *et al*,^17^ in patients with cancer, concluded that the fusogenic telomere length threshold provides a powerful, independent prognostic marker with clinical utility in breast cancer. Additionally, Needham *et al*^18^ examined the association between LTL and mortality in 3091 US adults aged 50–84 years, and concluded that the association between LTL and mortality differs by race/ethnicity and cause of death; the review performed by Haycock *et al*^19^ concluded that the available observational data show an inverse association between LTL and risk of coronary heart disease, independent of conventional vascular risk factors. The association with cerebrovascular disease is less certain. We have not found any study analysing the relationship of telomere length with the different factors involved in ageing in the Spanish population.

*Physical activity and vascular ageing*: There is ample evidence of the beneficial effect of exercise on vascular ageing.^20^ Regular aerobic physical activity of moderate intensity has been shown in adults to be associated with lower arterial stiffness and lower carotid intima-media thickness (IMT).^21^ ^22^ In contrast, in sedentary healthy adults, ageing is associated with increased stiffness of the large elastic arteries and with poorer vascular endothelial function and increased mortality.^22^ ^23^ The EVIDENT study found an association of physical activity with better vascular structure and function and sedentary time (hours spent watching television) with greater arterial stiffness.^24^ However, there are some points that are still unresolved such as the effect of endurance exercises, alone or combined with aerobic exercises, on vascular ageing.^22^ In addition, physical activity improves endothelial function, increases the bioavailability of nitric oxide and reduces the presence of oxidative stress and inflammatory agents, such as tumour necrosis factor-α (TNF-α).^25^ Therefore, physical activity favourably modulates several expressions of vascular ageing, thereby preserving vascular function and possibly reducing CVD risk.^1^ ^20^ ^26^

*Diet and vascular ageing*: The Mediterranean diet is currently considered the epitome of a healthy diet. There is evidence that it improves vascular ageing^27^ ^28^ and endothelial function,^29^ ^30^ increasing the number of endothelial cells and reducing vascular inflammation, oxidative stress and postprandial oxidative stress markers.^29^ ^30^ An omega-3 rich diet improves vascular ageing^27^ ^28^ and a caloric-restricted diet improves vascular structure and function.^31^ The PREDIMED study (Prevention with a Mediterranean Diet) has shown that participants in the intervention groups (Mediterranean diet) could reduce the incidence of major cardiovascular complications by 30% at 4 years of follow-up.^32^

*Psychological factors and vascular ageing*: Depression increases the risk of coronary disease (CD),^33^ predicts the increase in mortality after angioplasty or coronary surgery,^34^ ^35^ increases the risk of cerebrovascular disease,^36^ and increases mortality and the number of admissions in participants with heart failure (HF).^37^ ^38^

*Anxiety disorders* have also been associated with increased cardiac mortality and CD.^39^ In the INTERHEART study,^40^ participants who had a first myocardial infarction showed stress levels at work, stress at home, general and permanent stress greater than the controls. However, the association of psychological factors (depression, anxiety disorder and stress) with vascular stiffness and therefore with EVA is not well established.

*Smoking and vascular ageing*: Cigarette smoke stimulates leucocyte release of reactive oxygen and nitrogen species (ROS/RNS) and secretion of proinflammatory cytokines, increases the adherence of monocytes to the endothelium, and elicits airway inflammation. Smoking promotes endothelial dysfunction, decreasing nitric oxide and increasing oxidative stress.^5^ ^41^ Tobacco smoking enhances telomere shortening in circulating human white cell counts (WCCs). Telomere attrition (expressed in WCCs) can serve as a biomarker of the cumulative oxidative stress and inflammation induced by smoking and, consequently, show the pace of biological ageing.^42–44^

*Alcohol and vascular ageing*: In some studies, a beneficial effect of low or moderate alcohol consumption was found, particularly when taken as red wine.^9^ There are several studies that analysed the influence of alcohol in vascular stiffness and vascular ageing, concluding that moderate alcohol consumption may be beneficial.^45–47^

*Inflammatory markers and oxidative stress*: Decreased arterial endothelial function with age is an independent risk factor for the development of vascular disease in healthy adults.^1^ Similarly, microvascular dysfunction and vascular hyperpermeability, which alter the integrity of the endothelium and its barrier function, are promoted by oxidative stress, decreased nitric oxide, and proinflammatory changes and increased arterial stiffness.^3^ ^4^ ^48–51^

*MicroRNAs* (miRNA) have a critical role in the development of atherosclerotic disease, taking part in each step from the start of plaque formation to plaque destabilisation and rupture. Their analysis will help determine the pathophysiological mechanisms of arterial ageing.^52–56^

*Assessment of EVA*: Arterial stiffness can be assessed with different clinical tools. The currently accepted gold standard to assess arterial stiffness is the carotid-femoral pulse wave velocity (cf-PWV),^57^ ^58^ which is related to increased morbidity and mortality in patients with CVD and in healthy participants.^59^ The Cardio-Ankle Vascular Index (CAVI) is an index representing the stiffness of the aorta, femoral artery and tibial artery.^60^ Some authors suggest that CAVI, which is independent of blood pressure (BP) at the time of measurement, has an adequate reproducibility for clinical use, and is more useful as a marker of arterial stiffness than cf-PWV. It has also been observed that central BP is more related to cardiovascular morbidity and mortality than peripheral BP. The central and peripheral augmentation index (CAIx and PAIx) are also markers of arterial stiffness, which, together with the aortic systolic BP (SBP) and aortic pulse pressure, complement the information obtained with PWV.^57^ The presence of atherosclerosis can be measured with the IMT in the carotid artery and with the ankle-brachial index (ABI) or the amount of calcium in coronary arteries.

*Coordination and participants*: Eight research groups from the Biomedical Research Institute of Salamanca (IBSAL) have participated in this project, which will allow a multidisciplinary approach to the problem of EVA. The role of genetic factors and of the following exogenous factors will be analysed in a sample of the urban general population: all lifestyle aspects (toxic habits, diet, exercise), psychological factors, oxidative stress, inflammatory parameters and different parameters evaluating vascular structure and function.

*The general objectives of this study are*:

- To establish reference values for vascular structure and function in the population of Salamanca.
- To analyse the factors (physical, biological and psychological habits) influencing EVA, assessed by measuring the carotid-femoral pulse wave velocity (PWV) and cardio-ankle vascular index (CAVI).
- To determine differences by gender.

## Methods and analysis

### Study design

A cross-sectional, descriptive and observational study. We consider participants with EVA as those having cf-PWV and/or CAVI values greater than the 75th centile.

### Study setting

To be implemented in the Research Unit of La Alamedilla Primary Care Center, and on the premises of the hospital and University of Salamanca of the eight participating groups from the IBSAL.

### Study population

It will be the urban population attached to the Health Center of Salamanca. Using a random sampling stratified by age groups (35, 45, 55, 65 and 75 years) and gender, 100 participants will be selected in each group (50 males and 50 females), aged 35–75 years.

*Inclusion criteria*: Patients aged 35–75 years who agree to participate in the study and do not meet any of the exclusion criteria.

*Exclusion criteria*: Participants who are in terminal condition, who cannot travel to the health centres to undergo the corresponding examinations, and those who do not wish to sign the informed consent. Participants with a history of CVD (ischaemic heart disease or stroke, peripheral arterial disease or HF), diagnosed renal failure in terminal stages (glomerular filtration rate below 30%), chronic inflammatory disease or acute inflammatory process in the past 3 months. Patients treated with oestrogens, testosterone or growth hormone.

### Sample size

The sample size was estimated to find a difference of 0.60 points in the 14-item questionnaire on adherence to a Mediterranean diet between participants with EVA and those without. We used this variable because we think that it, of all the variables, varies the least over time. Assuming a 1:3 ratio between participants with and without EVA as the most unfavourable situation, accepting an α risk of 0.05 and a β risk of 0.20 in a two-sided test, and assuming a rate of losses due to technical difficulties or refusal to participate of 10%, 488 participants would be required, 122 in the first group and 366 in the second. It is assumed that the common SD is 1.94 points. Five hundred participants will be selected.

### Variables and measurement instruments

General and potentially effect-modifying variables such as age, gender, occupation, family and personal history of CVD and drug use will be documented. At the time of patient inclusion, we will record the date of diagnosis of hypertension or diabetes, as well as the drugs prescribed. We will also collect the start date of medication and doses of: antihypertensive, antidiabetic and antiplatelet agents; anticoagulants; and lipid-lowering drugs.

The occupation of the patient will be recorded using the National Classification of Occupations 2011 (CNO-11).^61^

### Anthropometric measurements

Body weight will be measured twice using a homologated electronic scale (Seca 770; Medical scale and measurement systems, Birmingham, UK) after calibration (precision±0.1 kg), with the patient wearing light clothing and barefoot. These readings will be rounded to 100 g. Height will be measured using a portable system (Seca 222; Medical scale and measurement systems, Birmingham, UK). The mean of two readings taken with the patient barefoot in the standing position will be recorded. Values will be rounded to the nearest centimetre. Body mass index (BMI) was calculated as weight (kg) divided by height squared. Waist circumference will be measured using a flexible graduated measuring tape with the patient in the standing position without clothing. The upper border of the iliac crests will be located, and the tape will be wrapped around above this point, parallel to the floor, ensuring that it is adjusted but without compressing the skin.

### Office or clinical BP

Office BP measurement will involve three measurements of SBP and diastolic BP (DBP), using the average of the last two, with a validated OMRON model M10-IT sphygmomanometer (Omron Health Care, Kyoto, Japan), by following the recommendations of the European Society of Hypertension.^62^ Pulse pressure will be estimated with the mean values of the second and third measurements.

### Habits and lifestyles

#### Diet

Adherence to the Mediterranean diet, principal end point of alimentation, will be measured using the validated 14-point Mediterranean Diet Adherence Screener (MEDAS),^63^ developed by the study group. MEDAS is a valid instrument for rapid estimation of adherence to the Mediterranean diet and may be useful in clinical practice. The 14-item screener includes 12 questions on food consumption frequency and two questions on food intake habits considered characteristic of the Spanish Mediterranean diet. Each question will be scored as 0 or 1. Adequate adherence to the Mediterranean diet will be assumed when the total score is ≥9 points.^63^ With the APP developed in the EVIDENT study (registry number 00/2014/2207), food consumption is recorded during a usual week. Data will be analysed with software developed for the analysis of food consumption in the EVIDENT II project.

#### Physical activity

*Actigraph GT3X accelerometers* (Actigraph, Shalimar, Florida, USA) will be used, which have been previously validated.^64^ Participants will wear the accelerometer fastened with an elastic strap to the right side of the waist for seven consecutive days, except while bathing and performing activities in the water. The data will be recorded at 1 min intervals. Total physical activity will be expressed in counts per minute. The intensity of physical activity will be determined according to the cut-off points proposed by Freedson,^65^ sedentary (<100 counts/min), light (100–1952 counts/min), moderate (1952–5724 counts/min), vigorous (>5724 counts/min) and very vigorous (>9498 counts/min). Moderate–vigorous activity will be considered as activity accumulated from all bouts lasting at least 1 min.

*The International Physical Activity Questionnaire—Short Form (IPAQ-SF)*: The short form (9 items) records the activity of four levels of intensity: (1) intense physical activity, such as aerobics, (2) moderate-intensity activity, such as leisure cycling, (3) walk and (4) sitting for 7 days.^66^ The data will be computed in METS/min/week.

*Questionnaire hours seated (Marshall)*: Evaluates the hours that the individual is sitting, in their work, in the displacements and at home, during the week and the weekend.^67^

*Paffenbarger Physical Activity Questionnaire*: Provides a complete representation of exercise. The questionnaire consists of eight questions. The following types of activity intensity are used: low (<4 mean metabolic equivalents (MET)), moderate (4 to <6 MET) and high (≥6 MET).^68–70^

#### Tobacco consumption

A questionnaire of four standard questions adapted from the WHO MONICA study will be used. The carbon monoxide concentration will be measured by a co-oximeter and the participants will be asked when the last cigarette was smoked.

#### Alcohol consumption

A questionnaire about alcohol consumption in the past 7 days will be included through a detailed questionnaire about alcohol types and volume.

##### Psychological factors

*Depression will be assessed with* *the Hamilton Depression (HAM-D)* *Scale*:^71^ The 17-item version was employed in the study. In this version, each item is scored from 0 to 2 or from 0 to 4; total scores can range from 0 to 52. The HAM-D scale was not originally designed with cut-off points to designate levels of severity of the depressive condition; we will define cut-off points and severity levels as follows: >23=very severe; 19–22=severe; 14–18=moderate; 8–13=mild and <7=remission.^72^ ^73^

*Anxiety will be assessed with the Hamilton Anxiety Scale*: The 17-item version will be used in the study. The severity of symptoms will be measured using five options of ordinal answer (0: no symptom; 4: very severe or disabling symptoms). The total score of the instrument, obtained by the sum of partial scores of 17 items, can vary within a range of 0 (no anxiety) to 68 (highest degree of anxiety) points.^74^ ^75^

*Stress will be assessed with Perceived Stress Scale Cohen* of 14 items. The scale scored from 0 to 56, where the upper scores indicate a higher perceived stress. It uses a Likert format with five alternatives ranging from 0 (‘never’) to 4 (‘always’).^76^

### Vascular structure and function

#### PWV and CAIx

These parameters will be estimated using the SphygmoCor System (AtCor Medical Pty Ltd, Head Office, West Ryde, Australia). With the patient sitting and resting his/her arm on a rigid surface, pulse wave analysis will be performed with a sensor in the radial artery, using a mathematical transformation to estimate the aortic pulse wave. CAIx will be estimated from aortic wave morphology using the following formula: increase in central pressure×100/pulse pressure, and it will be adjusted for heart rate at 75 bpm. Carotid and femoral artery pulse waves will be analysed, with the patient in a supine position, using the SphygmoCor System (Vx pulse wave velocity), estimating the delay as compared with the ECG wave and calculating PWV. Distance measurements will be taken with a measuring tape from the sternal notch to the carotid and femoral arteries at the sensor location and will be multiplied by 0.8. Subclinical organ damage of PWV will be defined as a cf-PWV>10 m/s.^77^

#### Cardio-ankle vascular index, brachial ankle PWV (ba-PWV) and ABI

These parameters will be estimated using the Vasera device VS-1500 (Fukuda Denshi). PWV will be calculated, as well as CAVI, which gives a more accurate estimation of the atherosclerosis degree. CAVI integrates cardiovascular elasticity derived from the aorta to the ankle pulse velocity through an oscillometric method; it is used as a good measure of vascular stiffness and does not depend on BP.^78^ CAVI values will be automatically calculated by substituting the stiffness parameters in the following equation to detect the vascular elasticity and the cardio ankle PWV: stiffness parameter β=2ρ×1/(Ps–Pd)×ln (Ps/Pd)×PWV^2^, where ρ is the blood density, Ps and Pd are SBP and DBP in mm Hg, and PWV is measured between the aortic valve and ankle. The average coefficient of the variation of CAVI is <5%, which is small enough for clinical use and confirms that CAVI has favourable reproducibility.^79^ CAVI and ABI will be measured in the resting position. ba-PWV is estimated using the following equation: ba-PWV=((0.5934×height (cm)+14.4724))/tba, where tba is the time the same waves were transmitted to the ankle.^80^

For the study, the lowest ABI and the highest CAVI and ba-PWV obtained will be considered. CAVI is classified as normal (CAVI<8), borderline (8≤CAVI<9) and abnormal (CAVI≥9). Abnormal CAVI represents subclinical atherosclerosis, and ba-PWV≥17.5 is considered abnormal.^81^ ^82^ ABI≤0.9 was considered abnormal.^45^

#### Assessment of vascular structure by carotid IMT (C-IMT)

Carotid ultrasound to assess C-IMT will be performed by two investigators trained for this purpose before starting the study. A Sonosite Micromaxx ultrasound device paired with a 5–10 MHz multifrequency high-resolution linear transducer with Sonocal software will be used for performing automatic measurements of C-IMT in order to optimise reproducibility. Measurements will be made of the common carotid after the examination of a 10 mm longitudinal section at a distance of 1 cm from the bifurcation, performing measurements in the proximal and in the distal wall in the lateral, anterior and posterior projections, following an axis perpendicular to the artery to discriminate two lines, one for the intima blood interface and the other for the media-adventitious interface. A total of six measurements will be obtained of the right carotid and another six of the left carotid, using average values (average C-IMT) and maximum values (maximum C-IMT) automatically calculated by the software.^83^ The measurements will be obtained with the participant lying down, with the head extended and slightly turned opposite to the examined carotid artery. The reliability was evaluated before the study began, using the intraclass correlation coefficient, which showed values of 0.974 (95% CI 0.935 to 0.990) for intraobserver agreement on repeated measurements in 20 participants, and 0.897 (95% CI 0.740 to 0.959) for interobserver agreement. According to the Bland-Altman analysis, the mean difference for intraobserver agreement (95% limits of agreement) was 0.022 (95% CI −0.053 to 0.098) and for interobserver agreement was 0.012 (95% CI −0.034 to 0.059). The average IMT will be considered abnormal if it measures >0.90 mm, or if there are atherosclerotic plaques with a diameter of 1.5 mm or a focal increase of 0.5 mm or 50% of the adjacent IMT.

#### Evaluation of retinal vessels

Retinography will be performed using a Topcon TRC NW 200 non-mydriatic retinal camera (Topcon Europe B.C., Capelle a/d Ijssel, The Netherlands), obtaining nasal and temporal images centred on the disk. The nasal image with the centred disk will be loaded into the developed software, AV Index calculator (Ciclorisk SL, Salamanca, Spain, registry number 00/2011/589). This software automatically recognises and draws two external concentric circles that delimit area A, 0–0.5 disk diameters from the optic disk margin, and area B, 0.5–1 disk diameters from the margin. The software first identifies the limits of the different vessels, after which it automatically recognises arteries and veins, and makes multiple measurements of the diameter of the section of vessels circulating through area B. It finally estimates the mean calibre of veins and arteries in millimetres, and these measurements are summarised as an arteriole–venule ratio, arteriovenous index (AVIx). An AVIx of 1.0 suggests that arteriolar diameters are on average the same as venular diameters in that eye, whereas a smaller arterio-venous ratio (AVR) suggests narrower arterioles. The pairs of main vessels in the upper and lower temporal quadrants will be used, rejecting all other vessels, to improve reliability and increase efficiency of the process, analysing measures for each quadrant separately and together to estimate the mean measure in each eye.

#### Coronary artery calcium score

Coronary calcium will be quantified using a 256-slice multidetector CT scanner (Brilliance iCT, Philips) according to the Agatston *et al*^84^ method, with the support of specific programming and specialised software (Intellispace Portal, Philips). This technology is the latest in the sector, allowing coronary calcium to be estimated with radiological exposures <1 mSv, equivalent to performing a spinal X-ray.

#### Renal assessment

Kidney damage will be assessed by measuring the estimated glomerular filtration rate using the Chronic Kidney Disease Epidemiology Collaboration (CKD-EPI)^85^ equation and proteinuria, as assessed by the albumin–creatinine ratio, following the criteria of the 2013 European Society of Hypertension/European Society of Cardiology Guidelines.^62^ Subclinical organ damage will be defined as glomerular filtration rate below 30–60 mL/min/1.73 m^2^ or microalbuminuria (30–300 mg/24 hours), or albumin–creatinine ratio (30–300 mg/g; 3.4–34 mg/mmol; preferably on morning spot urine). Renal disease will be defined as glomerular filtration rate <30 mL/min/1.73 m^2^ (body surface area), proteinuria (>300 mg/24 hours) or albumin–creatinine ratio >300 mg/24 hours.^62^

#### Cardiac assessment

ECG examination will be performed using a General Electric MAC 3.500 ECG System (Niskayuna, New York, USA), which automatically measures wave voltage and duration and estimates the criteria of the Cornell voltage-duration product (VDP).^86^ ECG left ventricular hypertrophy will be defined as a Sokolow-Lyon index >3.5 mV; R wave in Left arm (RaVL)>1.1 mV or Cornell VDP>244 mV×ms.^62^

#### Laboratory measurements

Venous blood sampling will be performed between 08:00 and 09:00 after participants have fasted and abstained from smoking and consumption of alcohol and caffeinated beverages for 12 hours. Fasting plasma glucose, creatinine, uric acid, Ca, P, serum total cholesterol, high-density lipoprotein cholesterol and triglyceride levels will be measured using standard enzymatic automated methods. Low-density lipoprotein cholesterol will be estimated by the Friedewald equation when the direct parameter is not available. Glycated haemoglobin will be measured with an immune-turbidimetric assay. Detection of 25-hydroxyvitamin D by immunoassay technical. High-sensitivity C reactive protein levels and fibrinogen concentrations will be determined by an immune turbidimetric assay. Cytokine (interleukin 1β, TNF-α) concentrations in plasma will be determined by ELISA using commercial kits and following the manufacturer's recommendations. Oxidative stress will be determined by measuring lipid peroxides (thiobarbituric acid reactive substances (TBARS), using a colorimetric technique using commercial kits) and nucleic acid derivatives (8-hydroxy-2-deoxyguanosine) by ELISA using commercial kits and following the manufacturer’s recommendations.

*Telomere length*: Telomere length will be measured using the real-time PCR technique. This is a technique that allows one to quantify the initial amount of DNA in a given fragment comparing this amplification with another performed simultaneously. To analyse these relative changes, a reference fragment in the DNA is chosen as the standard. In our study, we will use as the endogenous single copy control gene: 36b4. PCRs will be performed on 96-well optic plates (Micro Amp Fast Optical 96-Well reaction plate with Barcode 0.1 mL, Applied Biosystems). On each plate, two different reactions will be performed for each sample in different wells: an amplification reaction of telomeres and another amplification reaction of the 36b4 gene. In turn, we perform three replicates of each amplification to minimise the variability in concentration of the samples. In total, we will perform 9 PCRs for each patient. To amplify the 36b4 gene, we will use the primers: 36b4 F: 5′-CAGCAAGTGGGAAGGTGTAATCC-3′ and 36b4R: 5′-CCCATTCTATCATCAACGGGTACAA-3′; and to amplify the telomeres, we will use the primers TelF: 5′-GGTTTTTGAGGGTGAGGGTGAGGGTGAGGGTGAGGGT-3′ and TELR: 5′-TCCCGACTATCCCTATCCCTATCCCTATCCCTATCCCTATCCCTA-3′.

Blood samples will be collected in the primary care centre, and will be analysed at the University Hospital of Salamanca in external quality assurance programmes of the Spanish Society of Clinical Chemistry and Molecular Pathology.

People who perform the different tests will be blinded to the clinical patient data. All parameter assessments will be made within 10 days.

### Statistical analysis

Data input will be performed using the Teleform system (Autonomy Cardiff Vista, California, USA), with a questionnaire previously designed for the project. Normal distribution of variables will be verified using the Kolmogorov-Smirnov test. Quantitative variables will be displayed as mean±SD if normally distributed or as the median (IQR) if asymmetrically distributed, and qualitative variables will be expressed as frequencies. Analysis of difference of means between variables of two categories will be carried out using a Student’ t-test or a Mann-Whitney U test, as appropriate, while qualitative variables will be analysed using a χ² test. To analyse the relationship between qualitative variables of more than two categories and quantitative variables, an analysis of variance and the least significant difference test will be used in the post hoc tests; a Kruskal-Wallis test will be used in cases where the variables are not normally distributed. The relationship of quantitative variables to each other will be tested using Pearson or Spearman correlation as appropriate. Analysis of covariance (ANCOVA) will be performed to adjust for the variables that can affect the results as confounders. A multivariate analysis of variance (MANOVA) will be performed in cases with more than one dependent variable to identify whether changes in the independent variables have significant effects on the dependent variables. The association between the variables studied (PWV and CAVI with the independent variables analysed)) will be performed by multiple linear regression. Factors associated with the presence or absence of EVA will also be analysed by logistic regression. Data will be analysed using the SPSS V.23.0 statistical package (SPSS Inc, Chicago, Illinois, USA). A value of p<0.05 will be considered statistically significant. Statistical analysis of the extent of telomere length will be performed with the programs Step One 2.1 software (Applied Biosystems) and Enterprise GenEx 5.2.1.

To minimise the risk of bias resulting from foreseeable losses in the study, we will apply a process of multiple imputation to the original database, using the chained equations method proposed by van Buuren *et al*.^87^ Fifty files will be generated from this method, in which missing values will be replaced by their corresponding imputed values. Estimators obtained from each file will be combined by the method of Rubin.^88^ These processes will be carried out by the Ice^88^ and Mim programs, updated to V.14 of the statistical package Stata.

The statisticians/researchers who perform the different analyses will be blinded to the clinical patient data.

### Quality control

Different processes will be carried out to guarantee the study data quality and thus maximise the validity and reliability of measurements of the results. To this effect, field work operation manuals have been prepared. These documents specify the adequate procedure for performing each test. Educational leaflets will be developed to ensure adequate pressure measurement by patients at home. All of these actions will confirm adequate performance of each procedure. Monthly meetings will be held with the principal investigator of the study to analyse the entire process, and an annual report on study progress will be prepared.

#### Project schedule

This project will be performed in 3 years, with the aim to analyse factors that have an influence on EVA, estimated by cf-PWV and Cardio Ankle Vascular Index (CAVI), and to determine differences by gender in a Spanish population. During the first 2 years, the collection of data questionnaires, sample selection and inclusion should be developed and all the necessary information gathered. During the third year, the analysis and dissemination of the results will be performed.

## Dissemination

Participants will be required to sign an informed consent form prior to inclusion in the study, in accordance with the Declaration of Helsinki^89^ and the WHO standards for observational studies. The study has been registered in ClinicalTrials.gov with identifier NCT02623894. Participants will be informed of the objectives of the project and of the risks and benefits of the examinations made. None of the examinations pose life-threatening risks for the type of participants to be included in the study. The study includes the obtaining of biological samples; the study participants therefore will be informed in detail. The confidentiality of the recruited participants will be ensured at all times in accordance with the provisions of current legislation on personal data protection (15/1999 of 13 December Protection of Personal Data Official Law (LOPD)), and the conditions contemplated by Act 14/2007 on biomedical research.

### Dissemination plan

We will use a variety of methods to ensure that our work will achieve maximum visibility. Publication of our study protocol provides an important first step in this direction. In this paper, we have sought to offer a comprehensive overview of the relevant literature, while underlining current research gaps that necessitated the design and implementation of the EVA study.

Similarly, the study results, given their applicability and implications for the general population, will be disseminated in research meetings and in at least five articles published in scientific journals.

## Discussion

Numerous studies^5–9^ have analysed the factors influencing vascular stiffness, finding that arterial ageing is related to changes in the mechanical and structural properties of the vascular wall, which lead to loss of arterial elasticity and reduced distensibility. However, to what extent these changes occur through the action of environmental factors, lifestyles, psychological factors, inflammatory parameters and oxidative stress molecules predisposing to EVA has not been adequately studied. It is also not known which measure of arterial stiffness is best related to EVA.^40^ ^41^ ^43^ Thus, this study aims to provide evidence to clarify the influence and the relative weight of each of the factors on EVA, assessed using different tools measuring arterial ageing.^11^ ^12^

The benefits of moderate physical activity on arterial stiffness, and therefore on EVA, are well known.^15^ Similarly, the number of hours dedicated to sedentary activities is associated with increased stiffness of the large elastic arteries and poorer vascular endothelial function.^17^ However, the effect of endurance exercises, alone or combined with aerobic exercises, on vascular ageing is not clear.^17^

The Mediterranean diet,^27^ accompanied by caloric restriction, improved vascular structure and function.^26^ However, the dietary parameters associated with decreased EVA are not known.

Patients with depression,^28^ anxiety disorders^34^ or subjected to greater stress at work, stress at home, general stress and permanent stress^35^ have a greater risk of suffering CVD. However, we found no studies analysing the association of these factors with EVA. We hope this study will help clarify their influence on EVA.

It is known that smoking is harmful and that low or moderate alcohol consumption has a beneficial effect on CVD. Nevertheless, the weight they have on EVA has yet to be established.

We think that the project we are proposing has national and international relevance: the study of ageing and CVD has been established as a priority line of research in Europe and in Spain. To the best of our knowledge, there is no project in Spain or worldwide analysing in a population sample the majority of the potential factors currently known to influence EVA and attempting to quantify the weight and relevance of each of them. Therefore, we think that this is an original project and given the high prevalence of ageing of the population in our setting, a study to analyse the factors involved in arterial ageing will facilitate an understanding of this phenomenon and the degree of involvement of each factor in this process. In addition, it will provide knowledge for future research lines and potential clinical strategies.

### Study limitations

Although the study follows all the recommendations for observational studies considered in the STrengthening the Reporting of OBservational studies in Epidemiology (STROBE) statement, there is the possibility of confounding factors that have not been considered. The cross-sectional design of this study may lead us to observe an association, but not its direction over time.

*Strengths of the study*: The conduct of the study participants obtained by random and population-based sampling, with the involvement of eight research groups belonging to the areas of primary care, cardiovascular and gene and cell therapy of the IBSAL with extensive experience in the field, ensure the project's success. This study will create a platform and a specimen bank, as a basis for future studies, in which the role that miRNA plays in EVA will be analysed.

## References

[1] Villella E, Cho JS Effect of aging on the vascular system plus monitoring and support. Surg Clin North Am 2015;95:37–51. 10.1016/j.suc.2014.09.007 25459541

[2] Gragasin FS, Bourque SL, Davidge ST Vascular aging and hemodynamic stability in the intraoperative period. Front Physiol 2012;3:74 10.3389/fphys.2012.00074 22485091 PMC3317267

[3] Nilsson PM Hemodynamic aging as the consequence of structural changes associated with Early Vascular Aging (EVA). Aging Dis 2014;5:109–13. 10.14336/AD.2014.0500109 24729936 PMC3966669

[4] Oakley R, Tharakan B Vascular hyperpermeability and aging. Aging Dis 2014;5:114–25. 10.14336/AD.2014.0500114 24729937 PMC3966670

[5] Mitchell GF Effects of central arterial aging on the structure and function of the peripheral vasculature: implications for end-organ damage. J Appl Physiol 2008;105:1652–60. 10.1152/japplphysiol.90549.2008 18772322 PMC2584844

[6] Henry-Feugeas MC, Koskas P Cerebral vascular aging: extending the concept of pulse wave encephalopathy through capillaries to the cerebral veins. Curr Aging Sci 2012;5:157–67. 10.2174/1874609811205020157 22894741

[7] Lee HY, Oh BH Aging and arterial stiffness. Circ J 2010;74:2257–62. 10.1253/circj.CJ-10-0910 20962429

[8] Hashimoto J, Ito S Some mechanical aspects of arterial aging: physiological overview based on pulse wave analysis. Ther Adv Cardiovasc Dis 2009;3:367–78. 10.1177/1753944709338942 19574288

[9] O'Rourke MF, Hashimoto J Mechanical factors in arterial aging: a clinical perspective. J Am Coll Cardiol 2007;50:1–13. 10.1016/j.jacc.2006.12.050 17601538

[10] Maruyama Y Aging and arterial-cardiac interactions in the elderly. Int J Cardiol 2012;155:14–19. 10.1016/j.ijcard.2011.01.087 21316775

[11] Safar ME Arterial aging—hemodynamic changes and therapeutic options. Nat Rev Cardiol 2010;7:442–9. 10.1038/nrcardio.2010.96 20657613

[12] Najjar SS, Scuteri A, Lakatta EG Arterial aging: is it an immutable cardiovascular risk factor? Hypertension 2005;46:454–62. 10.1161/01.HYP.0000177474.06749.98 16103272

[13] Artandi SE Telomeres, telomerase, and human disease. N Engl J Med 2006;355:1195–7. 10.1056/NEJMp068187 16990382

[14] Marín C, Yubero-Serrano EM, López-Miranda J Endothelial aging associated with oxidative stress can be modulated by a healthy Mediterranean diet. Int J Mol Sci 2013;14:8869–89. 10.3390/ijms14058869 23615475 PMC3676761

[15] Rode L, Nordestgaard BG, Bojesen SE Peripheral blood leukocyte telomere length and mortality among 64,637 individuals from the general population. J Natl Cancer Inst 2015;107:djv074 10.1093/jnci/djv074 25862531

[16] Carty CL, Kooperberg C, Liu J Leukocyte telomere length and risks of incident coronary heart disease and mortality in a racially diverse population of postmenopausal women. Arterioscler Thromb Vasc Biol 2015;35:2225–31. 10.1161/ATVBAHA.115.305838 26249011 PMC4713196

[17] Simpson K, Jones RE, Grimstead JW Telomere fusion threshold identifies a poor prognostic subset of breast cancer patients. Mol Oncol 2015;9:1186–93. 10.1016/j.molonc.2015.02.003 25752197 PMC4449122

[18] Needham BL, Rehkopf D, Adler N Leukocyte telomere length and mortality in The National Health and Nutrition Examination Survey, 1999–2002. Epidemiology 2015;26:528–35. 10.1097/EDE.0000000000000299 26039272 PMC4679150

[19] Haycock PC, Heydon EE, Kaptoge S Leucocyte telomere length and risk of cardiovascular disease: systematic review and meta-analysis. BMJ 2014;349:g4227 10.1136/bmj.g4227 25006006 PMC4086028

[20] Gomez-Marcos MA, Recio-Rodríguez JI, Patino-Alonso MC Relationship between physical activity and plasma fibrinogen concentrations in adults without chronic diseases. PLoS ONE 2014;9:e87954 10.1371/journal.pone.0087954 24498413 PMC3912191

[21] Schmidt-Trucksass A, Weisser B [Vascular aging, arterial hypertension and physical activity]. Dtsch Med Wochenschr 2011;136:2367–71. 10.1055/s-0031-1292053 22068448

[22] Seals DR, Desouza CA, Donato AJ Habitual exercise and arterial aging. J Appl Physiol 2008;105:1323–32. 10.1152/japplphysiol.90553.2008 18583377 PMC2576026

[23] Jørgensen AB, Frikke-Schmidt R, Nordestgaard BG Loss-of-function mutations in APOC3 and risk of ischemic vascular disease. N Engl J Med 2014;371:32–41. 10.1056/NEJMoa1308027 24941082

[24] García-Ortiz L, Recio-Rodríguez JI, Schmidt-Trucksäss A Relationship between objectively measured physical activity and cardiovascular aging in the general population—the EVIDENT trial. Atherosclerosis 2014;233:434–40. 10.1016/j.atherosclerosis.2014.01.021 24530775

[25] Corbi G, Conti V, Russomanno G Is physical activity able to modify oxidative damage in cardiovascular aging? Oxid Med Cell Longev 2012;2012:728547 10.1155/2012/728547 23029599 PMC3458405

[26] Seals DR, Jablonski KL, Donato AJ Aging and vascular endothelial function in humans. Clin Sci 2011;120:357–75. 10.1042/CS20100476 21244363 PMC3482987

[27] Strazhesko ID, Akasheva DU, Dudinskaia EN [The renin-angiotensin-aldosterone system and vascular aging]. Kardiologiia 2013;53:78–84. 24087966

[28] Patino-Alonso MC, Recio-Rodríguez JI, Belio JF Factors associated with adherence to the Mediterranean diet in the adult population. J Acad Nutr Diet 2014;114:583–9. 10.1016/j.jand.2013.07.038 24209889

[29] Scuteri A, Cunha PG Decreasing arterial aging by controlling blood pressure levels and hypertension: a step forward. Curr Vasc Pharmacol 2012;10:702–4. 10.2174/157016112803520954 23259559

[30] Vlachopoulos C Progress towards identifying biomarkers of vascular aging for total cardiovascular risk prediction. J Hypertens 2012;30(Suppl):S19–26. 10.1097/HJH.0b013e328353e534 23124101

[31] Khurana S, Venkataraman K, Hollingsworth A Polyphenols: benefits to the cardiovascular system in health and in aging. Nutrients 2013;5:3779–827. 10.3390/nu5103779 24077237 PMC3820045

[32] Estruch R, Ros E, Salas-Salvadó J, PREDIMED Study Investigators. Primary prevention of cardiovascular disease with a Mediterranean diet. N Engl J Med 2013;368:1279–90. 10.1056/NEJMoa1200303 29897867

[33] Lespérance F, Frasure-Smith N, Talajic M Five-year risk of cardiac mortality in relation to initial severity and one-year changes in depression symptoms after myocardial infarction. Circulation 2002;105:1049–53. 10.1161/hc0902.104707 11877353

[34] Blumenthal JA, Lett HS Babyak MA Depression as a risk factor for mortality after coronary artery bypass surgery. Lancet 2003;362:604–9. 10.1016/S0140-6736(03)14190-6 12944059

[35] Nicholson A, Kuper H, Hemingway H Depression as an aetiologic and prognostic factor in coronary heart disease: a meta-analysis of 6362 events among 146 538 participants in 54 observational studies. Eur Heart J 2006;27:2763–74. 10.1093/eurheartj/ehl338 17082208

[36] Van der Kooy K, van Hout H, Marwijk H Depression and the risk for cardiovascular diseases: systematic review and meta analysis. Int J Geriatr Psychiatry 2007;22:613–26. 10.1002/gps.1723 17236251

[37] Sherwood A, Blumenthal JA, Trivedi R Relationship of depression to death or hospitalization in patients with heart failure. Arch Intern Med 2007;167:367–73. 10.1001/archinte.167.4.367 17325298

[38] Lesman-Leegte I, van Veldhuisen DJ, Hillege HL Depressive symptoms and outcomes in patients with heart failure: data from the COACH study. Eur J Heart Fail 2009;11:1202–7. 10.1093/eurjhf/hfp155 19926602

[39] Janszky I, Ahnve S, Lundberg I Early-onset depression, anxiety, and risk of subsequent coronary heart disease: 37-year follow-up of 49,321 young Swedish men. J Am Coll Cardiol 2010;56:31–7. 10.1016/j.jacc.2010.03.033 20620714

[40] Rosengren A, Hawken S, Ounpuu S Association of psychosocial risk factors with risk of acute myocardial infarction in 11119 cases and 13648 controls from 52 countries (the INTERHEART study): case-control study. Lancet 2004;364:953–62. 10.1016/S0140-6736(04)17019-0 15364186

[41] Csiszar A, Podlutsky A, Wolin MS Oxidative stress and accelerated vascular aging: implications for cigarette smoking. Front Biosci (Landmark Ed) 2009;14:3128–44. 19273262 10.2741/3440 PMC2756477

[42] Pietri P, Vlachopoulos C, Chrysohoou C Deceleration of age-related aortic stiffening in a population with high longevity rates: the IKARIA study. J Am Coll Cardiol 2015;66:1842–3. 10.1016/j.jacc.2015.07.070 26483111

[43] Babizhayev MA, Yegorov YE Smoking and health: association between telomere length and factors impacting on human disease, quality of life and life span in a large population-based cohort under the effect of smoking duration. Fundam Clin Pharmacol 2011;25:425–42. 10.1111/j.1472-8206.2010.00866.x 20698892

[44] Babizhayev MA, Savel'yeva EL, Moskvina SN Telomere length is a biomarker of cumulative oxidative stress, biologic age, and an independent predictor of survival and therapeutic treatment requirement associated with smoking behavior. Am J Ther 2011;18:e209–26. 10.1097/MJT.0b013e3181cf8ebb 20228673

[45] Karimi L, Mattace-Raso FU, van Rosmalen J Effects of combined healthy lifestyle factors on functional vascular aging: the Rotterdam Study. J Hypertens 2016;34:853–9. 10.1097/HJH.0000000000000861 26882039

[46] Tyrovolas S, Haro JM, Polychronopoulos E Factors associated with components of arterial pressure among older individuals (the multinational MEDIS study): the role of the Mediterranean diet and alcohol consumption. J Clin Hypertens (Greenwich) 2014;16:645–51. 10.1111/jch.12370 25056587 PMC8031500

[47] Mattace-Raso FU, van der Cammen TJ, van den Elzen AP Moderate alcohol consumption is associated with reduced arterial stiffness in older adults: the Rotterdam study. J Gerontol A Biol Sci Med Sci 2005;60:1479–83. 16339338 10.1093/gerona/60.11.1479

[48] Lakatta EG, Wang M, Najjar SS Arterial aging and subclinical arterial disease are fundamentally intertwined at macroscopic and molecular levels. Med Clin North Am 2009;93:583–604, Table of Contents 10.1016/j.mcna.2009.02.008 19427493 PMC2943242

[49] Sun Z Aging, arterial stiffness, and hypertension. Hypertension 2015;65:252–6. 10.1161/HYPERTENSIONAHA.114.03617 25368028 PMC4288978

[50] Kim JY, Kim OY, Paik JK Association of age-related changes in circulating intermediary lipid metabolites, inflammatory and oxidative stress markers, and arterial stiffness in middle-aged men. Age (Dordr) 2013;35:1507–19. 10.1007/s11357-012-9454-2 22806411 PMC3705113

[51] Bachschmid MM, Schildknecht S, Matsui R Vascular aging: chronic oxidative stress and impairment of redox signaling-consequences for vascular homeostasis and disease. Ann Med 2013;45:17–36. 10.3109/07853890.2011.645498 22380696 PMC3717565

[52] Menghini R, Stohr R, Federici M MicroRNAs in vascular aging and atherosclerosis. Ageing Res Rev 2014;17:68–78. 10.1016/j.arr.2014.03.005 24681293

[53] Nyholm AM, Lerche CM, Manfé V miR-125b induces cellular senescence in malignant melanoma. BMC Dermatol 2014;14:1471–5945. 10.1186/1471-5945-14-8 PMC4021480 24762088

[54] van Balkom BW, de Jong OG, Smits M Endothelial cells require miR-214 to secrete exosomes that suppress senescence and induce angiogenesis in human and mouse endothelial cells. Blood 2013;121:3997–4006, S1–15 10.1182/blood-2013-02-478925 23532734

[55] Fenn AM, Smith KM, Lovett-Racke AE Increased micro-RNA 29b in the aged brain correlates with the reduction of insulin-like growth factor-1 and fractalkine ligand. Neurobiol Aging 2013;34:2748–58. 10.1016/j.neurobiolaging.2013.06.007 23880139 PMC3779520

[56] Xu C, Yamamoto-Ibusuki M, Yamamoto Y High survivin mRNA expression is a predictor of poor prognosis in breast cancer: a comparative study at the mRNA and protein level. Breast Cancer 2014;21:482–90. 10.1007/s12282-012-0403-9 22968628

[57] Mattace-Raso FU, van der Cammen TJ, Hofman A Arterial stiffness and risk of coronary heart disease and stroke: the Rotterdam Study. Circulation 2006;113:657–63. 10.1161/CIRCULATIONAHA.105.555235 16461838

[58] Williams B, Lacy PS, Thom SM Differential impact of blood pressure-lowering drugs on central aortic pressure and clinical outcomes: principal results of the Conduit Artery Function Evaluation (CAFE) study. Circulation 2006;113:1213–25. 10.1161/CIRCULATIONAHA.105.595496 16476843

[59] Laurent S Defining vascular aging and cardiovascular risk. J Hypertens 2012;30(Suppl):S3–8. 10.1097/HJH.0b013e328353e501 23124102

[60] Shirai K, Utino J, Saiki A Evaluation of blood pressure control using a new arterial stiffness parameter, cardio-ankle vascular index (CAVI). Curr Hypertens Rev 2013;9:66–75. 10.2174/1573402111309010010 23807874 PMC3636518

[61] INE. *Clasificación Nacional de Ocupaciones 2011 (CNO-11) URL* 2011 http://wwwinees/jaxi/menudo?type=pcaxis&path=/t40/cno11&file=inebase,.

[62] Mancia G, Fagard R, Narkiewicz K 2013 ESH/ESC Guidelines for the management of arterial hypertension: the Task Force for the management of arterial hypertension of the European Society of Hypertension (ESH) and of the European Society of Cardiology (ESC). J Hypertens 2013;31:1281–357. 10.1097/01.hjh.0000431740.32696.cc 23817082

[63] Schroder H, Fito M, Estruch R A short screener is valid for assessing Mediterranean diet adherence among older Spanish men and women. J Nutr 2011;141:1140–5. 10.3945/jn.110.135566 21508208

[64] Melanson EL Jr, Freedson PS Validity of the Computer Science and Applications, Inc. (CSA) activity monitor. Med Sci Sports Exerc 1995;27:934–40. 7658958

[65] Freedson PS, Melanson E, Sirard J Calibration of the Computer Science and Applications, Inc. accelerometer. Med Sci Sports Exerc 1998;30:777–81. 10.1097/00005768-199805000-00021 9588623

[66] Craig CL, Marshall AL, Sjöström M International physical activity questionnaire: 12-country reliability and validity. Med Sci Sports Exerc 2003;35:1381–95. 10.1249/01.MSS.0000078924.61453.FB 12900694

[67] Marshall AL, Miller YD, Burton NW Measuring total and domain-specific sitting: a study of reliability and validity. Med Sci Sports Exerc 2010;42:1094–102. 10.1249/MSS.0b013e3181c5ec18 19997030

[68] Nowak Z, Plewa M, Skowron M Paffenbarger Physical Activity Questionnaire as an additional tool in clinical assessment of patients with coronary artery disease treated with angioplasty. Kardiol Pol 2010;68:32–9. 20131186

[69] Simpson K, Parker B, Capizzi J Validity and reliability question 8 of the Paffenbarger Physical Activity Questionnaire among healthy adults. J Phys Act Health 2015;12:116–23. 10.1123/jpah.2013-0013 24733349

[70] Paffenbarger RS Jr, Blair SN, Lee IM Measurement of physical activity to assess health effects in free-living populations. Med Sci Sports Exerc 1993;25:60–70. 10.1249/00005768-199301000-00010 8423758

[71] Hamilton M A rating scale for depression. J Neurol Neurosurg Psychiatr 1960;23:56–62. 10.1136/jnnp.23.1.56 PMC495331 14399272

[72] Ramos-Brieva JA, Cordero Villafafila A [Validation of the Castillian version of the Hamilton Rating Scale for Depression]. Actas Luso Esp Neurol Psiquiatr Cienc Afines 1986;14:324–34. 3776732

[73] Montgomery SA, Asberg M A new depression scale designed to be sensitive to change. Br J Psychiatry 1979;134:382–9. 10.1192/bjp.134.4.382 444788

[74] Hamilton M The assessment of anxiety states by rating. Br J Med Psychol 1959;32:50–5. 10.1111/j.2044-8341.1959.tb00467.x 13638508

[75] Lobo A, Chamorro L, Luque A [Validation of the Spanish versions of the Montgomery-Asberg depression and Hamilton anxiety rating scales]. Med Clin (Barc) 2002;118:493–9. 10.1016/S0025-7753(02)72429-9 11975886

[76] Cohen S, Kamarck T, Mermelstein R A global measure of perceived stress. J Health Soc Behav 1983;24:385–96. 10.2307/2136404 6668417

[77] Van Bortel LM, Laurent S, Boutouyrie P Expert consensus document on the measurement of aortic stiffness in daily practice using carotid-femoral pulse wave velocity. J Hypertens 2012; 30:445–8. 10.1097/HJH.0b013e32834fa8b0 22278144

[78] Shirai K, Hiruta N, Song M Cardio-ankle vascular index (CAVI) as a novel indicator of arterial stiffness: theory, evidence and perspectives. J Atheroscler Thromb 2011;18:924–38. 10.5551/jat.7716 21628839

[79] Shirai K, Utino J, Otsuka K A novel blood pressure-independent arterial wall stiffness parameter; cardio-ankle vascular index (CAVI). J Atheroscler Thromb 2006;13:101–7. 10.5551/jat.13.101 16733298

[80] Yamashina A, Tomiyama H, Takeda K Validity, reproducibility, and clinical significance of noninvasive brachial-ankle pulse wave velocity measurement. Hypertens Res 2002;25:359–64. 10.1291/hypres.25.359 12135313

[81] Kawai T, Ohishi M, Onishi M Cut-off value of brachial-ankle pulse wave velocity to predict cardiovascular disease in hypertensive patients: a cohort study. J Atheroscler Thromb 2013;20:391–400. 10.5551/jat.15040 23268984

[82] Korkmaz L, Erkan H, Korkmaz AA Relationship of aortic knob width with cardio-ankle vascular stiffness index and its value in diagnosis of subclinical atherosclerosis in hypertensive patients: a study on diagnostic accuracy. Anadolu Kardiyol Derg 2012;12:102–6. 10.5152/akd.2012.034 22281788

[83] Gómez-Marcos MA, Recio-Rodríguez JI, Patino-Alonso MC Protocol for measuring carotid intima-media thickness that best correlates with cardiovascular risk and target organ damage. Am J Hypertens 2012;25:955–61. 10.1038/ajh.2012.72 22717546

[84] Agatston AS, Janowitz WR, Hildner FJ Quantification of coronary artery calcium using ultrafast computed tomography. J Am Coll Cardiol 1990;15:827–32. 10.1016/0735-1097(90)90282-T 2407762

[85] Levey AS, Stevens LA, Schmid CH A new equation to estimate glomerular filtration rate. Ann Intern Med 2009;150:604–12. 10.7326/0003-4819-150-9-200905050-00006 19414839 PMC2763564

[86] Okin PM, Roman MJ, Devereux RB Electrocardiographic identification of increased left ventricular mass by simple voltage-duration products. J Am Coll Cardiol 1995;25:417–23. 10.1016/0735-1097(94)00371-V 7829796

[87] van Buuren S, Boshuizen HC, Knook DL Multiple imputation of missing blood pressure covariates in survival analysis. Stat Med 1999;18:681–94. 10204197 10.1002/(sici)1097-0258(19990330)18:6<681::aid-sim71>3.0.co;2-r

[88] Li KH, Raghunathan TE, Rubin DB Large-sample significance levels from multiply imputed data using moment-based statistics and an F reference distribution. J Am Stat Assoc 1991;86:1063–73.

[89] World Medical Association. World Medical Association Declaration of Helsinki: ethical principles for medical research involving human subjects. JAMA 2013;310:2191–4. 10.1001/jama.2013.281053 24141714

